# Gastric Perforation as a Complication of COVID-19 Infection: A Case Report

**DOI:** 10.7759/cureus.23725

**Published:** 2022-04-01

**Authors:** Abdulrahman S Almulhim, Alaa Alghazzi, Ali A Almohammed saleh, Ahmed H Alsulaiman, Lojain A Alnosair, Fatimah Y Alghareeb

**Affiliations:** 1 General Surgery, King Faisal University, Al-Ahsa, SAU; 2 Surgery, King Fahad General Hospital, Hofuf, SAU; 3 Medicine, King Faisal University, Al-Ahsa, SAU; 4 Medicine, Imam Abdulrahman Bin Faisal University, Dammam, SAU; 5 General Surgery, King Fahad General Hospital, Al-Ahsa, SAU

**Keywords:** sars-cov 2 infection, medical icu, multi-organ dysfunction, covid 19, gastrointestinal perforation

## Abstract

Pulmonary symptoms are the primary manifestation of the COVID-19 disease, which originated in Wuhan in China in December 2019. However, it is now established to show widespread extrapulmonary manifestations, including gastrointestinal involvement. Abdominal pain, diarrhea, nausea, and vomiting are considered the most common gastrointestinal symptoms. Gastric perforation in association with COVID-19 is rarely reported in the literature. Here, we report a case of a 71-year-old male COVID-19-infected patient, medically free, who presented to the emergency department complaining of severe abdominal pain for a one-week duration. He was diagnosed with a case of perforated viscus and septic shock. The patient was shifted to the operating room for exploratory laparotomy. We aim in this report to highlight this fatal complication of COVID-19 infection in order to improve patients' outcomes.

## Introduction

Coronavirus disease 2019 (COVID-19), which is caused by the SARS-CoV-2 virus, has rapidly spread, causing a worldwide pandemic since 2020. Thorough comprehension of COVID-19 and its complications is still under research [1]. Although the typical presentation of COVID-19 revolves around acute respiratory symptoms, extra-pulmonary manifestations have been acknowledged [2,3]. COVID-19 is associated with cardiac, renal, neurological, and gastrointestinal manifestations [3]. Even though the common gastrointestinal features of COVID-19 are abdominal pain, nausea, vomiting, and diarrhea, several reported cases suggested an association between COVID-19 and viscus perforation [1,4,5]. We hereby present a case report of a 71-year-old patient who was COVID-19 positive when he developed viscus perforation. The perforation was at the site of a gastric anastomosis of a biliopancreatic diversion surgery (BPD) that was performed 14 years ago. BPD is a bariatric procedure that was widely popular in the 90s but is not recommended anymore due to its wide range of complications, including stomal ulcers [6,7].

## Case presentation

A 71-year-old male patient, medically free, presented to the emergency department complaining of severe abdominal pain for a one-week duration. The abdominal pain was generalized, not radiating to any site, and associated with vomiting, hematemesis, and melena. During history taking, the patient revealed that he is COVID-19 positive since 12 days prior to presentation. He is a non-smoker, non-alcoholic, and has no history of drug abuse. Surgical history revealed a previous biliopancreatic diversion surgery which he underwent 14 years ago. He also had a hip fixation surgery five months ago, for which he used non-steroidal anti-inflammatory drugs (NSAIDs) for a one-week duration. His vital signs showed a temperature of 36.9 C, heart rate of 102 bpm, blood pressure of 80/45 mm Hg, and peripheral oxygen saturation (spO2) of 92% in the room air.

**Figure 1:**
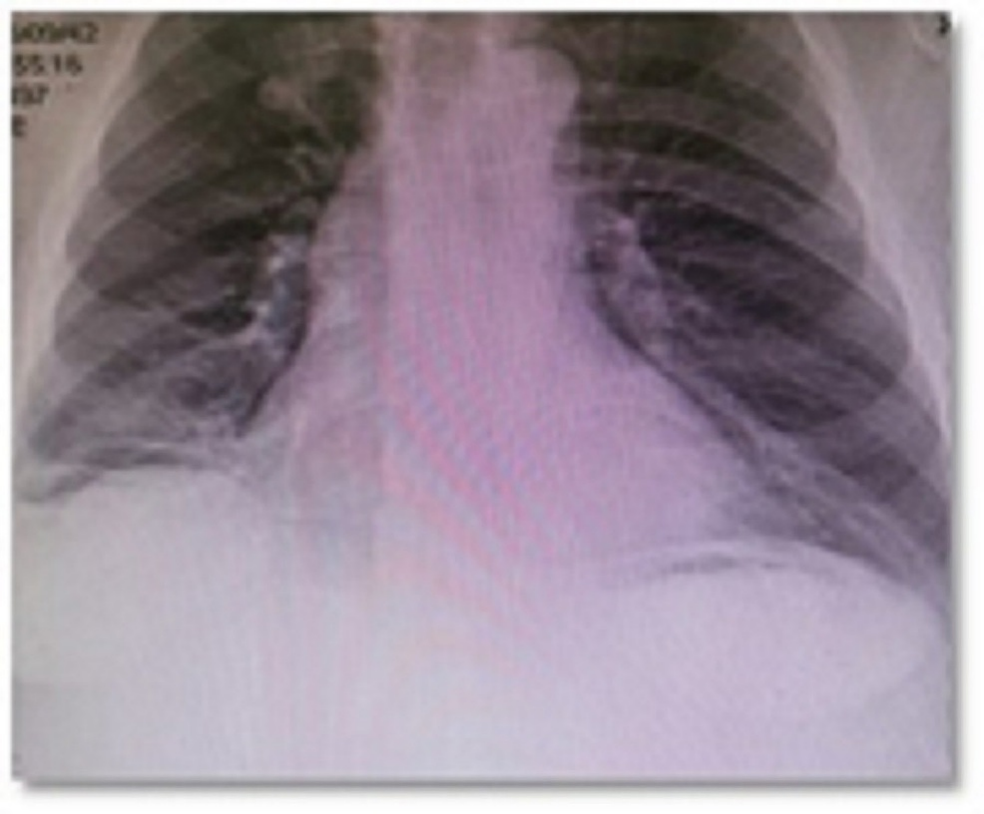
Chest X-ray showing pneumoperitoneum suggesting viscus perforation

On physical examination, the patient was conscious, alert, and oriented to time, place, and person. His abdomen was distended with generalized tenderness. Laboratory investigations showed the following: white blood cells (WBCs) 36 (normal range is 4-10), hemoglobin (Hb) 9.1 (normal range is 13-17), prothrombin time (PT) 22.2 (normal range is 9.8-13.2), creatinine 212 (normal range is 46-110), blood urea nitrogen (BUN) 22 (normal range is 3.2-7.1), international normalized ratio (INR) 1.6 (normal range is 0.9-1.2), total bilirubin 33.3 (normal range is 3-22). Pneumoperitoneum was seen on a chest X-ray, which suggested bowel perforation (Figure 1).

Intravenous fluid (IVF) boluses, omeprazole infusions, and three units of fresh frozen plasma transfusions were used to resuscitate the patient. He was admitted as a case of perforated viscus and septic shock. He was shifted to the operating room for an exploratory laparotomy. Under general anesthesia, a midline incision was done. A perforated bleeding ulcer measuring approximately 3x3 cm was discovered at the prior gastric anastomosis site with pus collection. A nasogastric tube was placed beyond the level of perforation. Several interrupted vicryl stitches and a Graham patch were performed to repair the defect. Peritoneal toileting was carried out, and hemostasis was secured. Two drains were fixed, the left one at the pelvis and the right one at the repair site.

**Figure 2:**
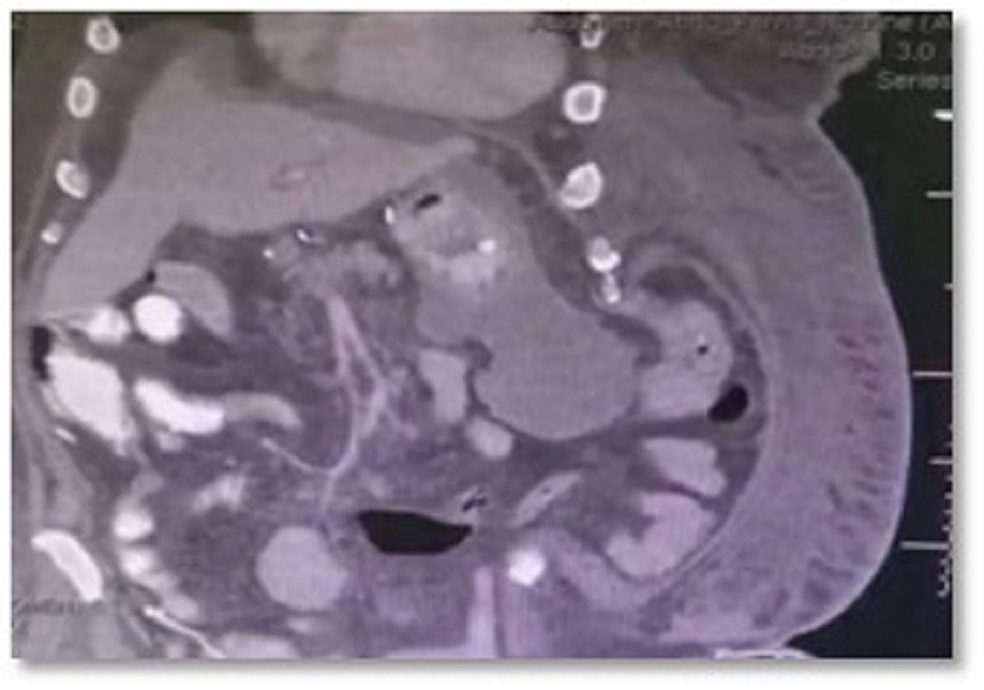
A CT scan of the abdomen and pelvis A CT scan of the abdomen and pelvis with oral contrast showing fluid accumulation in the left upper abdomen, related to the stomach and extending inferiorly. It measured about 5.7 x 8.2 x 9.8 cm in size. A fluid collection of 6.9 x 7.3 x 1.7 cm was also found superior to the left hepatic lobe. There was no CT evidence of intra-abdominal free air or contrast leak.

**Figure 3:**
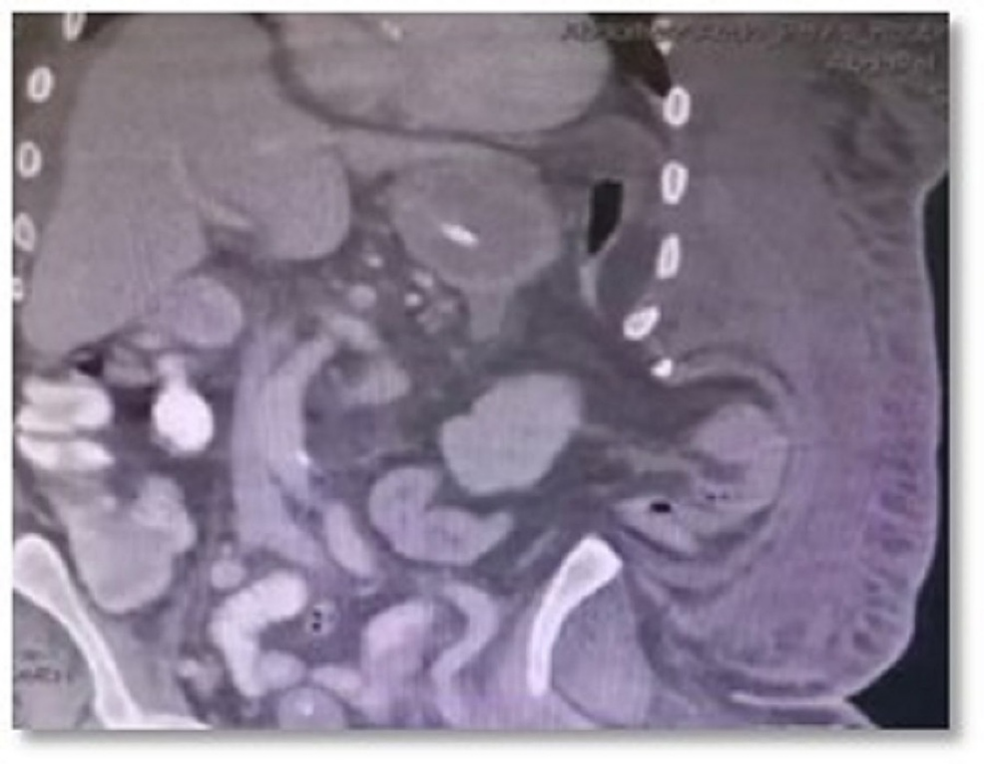
A CT scan of the abdomen and pelvis A CT scan of the abdomen and pelvis with oral contrast showing fluid accumulation in the left upper abdomen, related to the stomach and extending inferiorly. It measured about 5.7 x 8.2 x 9.8 cm in size. A fluid collection of 6.9 x 7.3 x 1.7 cm was also found superior to the left hepatic lobe. There was no CT evidence of intra-abdominal free air or contrast leak.

The patient was admitted into the COVID-19 intensive care unit (ICU) isolation, where he developed multiorgan failure and disseminated intravascular coagulation (DIC). A complete blood count (CBC) was done and revealed a very high level of WBC (41.89) and Hb of 9.3. INR was 1.33, and PT was 18.6. The patient was given noradrenaline and started on continuous renal replacement therapy. The patient remained in the ICU for 10 days, until the COVID-19 swab test became negative, and was shifted to the surgical ward afterward. He was able to tolerate nasogastric tube (NGT) feeding and received daily wound dressings. Computed tomography (CT) scan of the abdomen and pelvis with oral contrast was carried out, revealing a fluid accumulation in the left upper abdomen (Figures 2, 3).

The patient went into cardiac arrest in the surgical ward and was revived after five minutes of cardiopulmonary resuscitation (CPR). He was intubated under anesthesia and admitted back into the ICU. The patient improved after one week and thus was extubated moved back to the surgical ward. He was kept on nasogastric tube feeding with good toleration and good bowel motion. On day 26 post-operation, his condition began to deteriorate; he desaturated down to 90% spO2 on room air with hypotension. As a result, he was transferred to ICU, where he died on the fourth day due to cardiopulmonary arrest.

## Discussion

Since the beginning of the COVID-19 pandemic, which initially developed in China, many studies have revealed that respiratory symptoms are the most common representing manifestations of this infection, which include fever, dyspnea, and cough [8]. However, the prevalence of gastrointestinal manifestations of COVID-19 is 10% to 50% [9,10]. So, gastrointestinal manifestations are the most frequently reported extra-pulmonary manifestations [11]. Abdominal pain, diarrhea, nausea, and vomiting are the most commonly reported gastrointestinal symptoms associated with the COVID-19 infection [9-11]. Moreover, there have been several articles that reported COVID-19 positive patients presenting with different gastrointestinal manifestations. These include acute surgical abdomen [12], acute pancreatitis [13], and lower gastrointestinal bleeding [14]. It has been shown that the gastrointestinal tract is a common site of infection for SARS-CoV-2. The mechanism of infection in the gastrointestinal tract is the high expression of the angiotensin-converting enzyme (ACE) 2 receptor. SARS-CoV-2 then starts to bind to this receptor in order to get inside the host cells [15]. The epithelium of the stomach, the duodenum, and the rectum were found to have the highest staining of this receptor [16]. In a systemic review and meta-analysis that was done by Henry et al., it was found that the severity of gastrointestinal symptoms may be correlated to the severity of the COVID-19 infection [17]. There are minimal articles that reported gastrointestinal (GI) perforation as a result of COVID-19 infection. GI perforation is a surgical emergency with a very high risk of mortality due to peritonitis that could be complicated by multi-organ failure [18]. Gonzálvez Guardiola et al. reported a case of a positive COVID-19 male patient diagnosed with a cecum perforation that was treated with right colectomy [19]. Our patient presented with a perforation at the previous gastric anastomosis site following a previous biliopancreatic diversion procedure. Similarly, Galvez et al. reported a case of an elderly female COVID-19 positive patient with a previous laparoscopic Roux-en-Y gastric bypass surgery presented with acute abdominal pain and worsening dyspnea. She was diagnosed with a GI perforation at the gastro-jejunal anastomosis site [20].

Biliopancreatic diversion (BPD) is an operation that has been performed on morbidly obese patients. It consists of a distal gastrectomy with a long Roux-en-Y reconstruction [21]. Even though surgical complications following BPD are rare, they require urgent attention and immediate management. These complications include hemorrhage and perforation [22]. Anderson et al. reported a case of a female patient post BPD 13 months back, diagnosed with gastric perforation at the distal BPD jejunoileal anastomosis [23]. Cossu et al. reported a case of gastric perforation with a history of BPD that was performed five years back [22]. However, a gastric perforation after a long period of BPD (14 years back) has not been reported in the literature, which is another interesting point regarding our patient presentation. In addition, our patient was at higher risk of gastric perforation due to the recent NSAIDs usage, as these medications could directly or indirectly irritate the intestinal mucosa and lead to the risk of gastric perforation [24]. However, our patient used NSAIDs for a short time (one week) and a long time before the hospitalization. 

A typical history of gastric perforation and radiographic evidence of pneumo-peritoneum are often sufficient for diagnosis [25]. Surgical management is the mainstay of treatment in patients with gastric perforation in order to improve survival [26]. However, in the absence of peritonitis signs or abdominal sepsis, conservative treatment is acceptable [27,28]. Our patient was diagnosed with a case of perforated viscus and septic shock. We managed him surgically, but unfortunately, he developed multi-organ failure and died on the 26th postoperative day. 

## Conclusions

Gastrointestinal manifestations were frequently reported in patients with COVID-19. However, gastric perforation is rarely reported in the literature. Gastric perforation is an important differential for patients presenting with sudden severe abdominal pain. The coincidence of COVID-19 infection and emergency surgical pathology such as a perforated ulcer presents a unique challenge to clinicians. Understanding the gastrointestinal correlation to COVID-19 and the associated morbidities in the adult population is crucial in order to avoid fatal complications.

## References

[1] Hu B, Guo H, Zhou P, Shi ZL (2021). Characteristics of SARS-CoV-2 and COVID-19. Nat Rev Microbiol.

[2] Lai CC, Shih TP, Ko WC, Tang HJ, Hsueh PR (2020). Severe acute respiratory syndrome coronavirus 2 (SARS-CoV-2) and coronavirus disease-2019 (COVID-19): the epidemic and the challenges. Int J Antimicrob Agents.

[3] Lai CC, Ko WC, Lee PI, Jean SS, Hsueh PR (2020). Extra-respiratory manifestations of COVID-19. Int J Antimicrob Agents.

[4] Lin L, Jiang X, Zhang Z (2020). Gastrointestinal symptoms of 95 cases with SARS-CoV-2 infection. Gut.

[5] De Nardi P, Parolini DC, Ripa M, Racca S, Rosati R (2020). Bowel perforation in a Covid-19 patient: case report. Int J Colorectal Dis.

[6] Michielson D, Van Hee R, Hendrickx L (1996). Complications of biliopancreatic diversion surgery as proposed by Scopinaro in the treatment of morbid obesity. Obes Surg.

[7] Totté E, Hendrickx L, van Hee R (1999). Biliopancreatic diversion for treatment of morbid obesity: experience in 180 consecutive cases. Obes Surg.

[8] Zhu N, Zhang D, Wang W (2020). A Novel Coronavirus from Patients with Pneumonia in China, 2019. N Engl J Med.

[9] Pan L, Mu M, Yang P (2020). Clinical characteristics of COVID-19 patients with digestive symptoms in Hubei, China: a descriptive, cross-sectional, multicenter study. Am J Gastroenterol.

[10] Rokkas T (2020). Gastrointestinal involvement in COVID-19: a systematic review and meta-analysis. Ann Gastroenterol.

[11] Guan WJ, Ni ZY, Hu Y (2020). Clinical characteristics of coronavirus disease 2019 in China. N Eng J Med.

[12] Ashcroft J, Hudson VE, Davies RJ (2020). COVID-19 gastrointestinal symptoms mimicking surgical presentations. Ann Med Surg (Lond).

[13] Hadi A, Werge M, Kristiansen KT, Pedersen UG, Karstensen JG, Novovic S, Gluud LL (2020). Coronavirus disease-19 (COVID-19) associated with severe acute pancreatitis: case report on three family members. Pancreatology.

[14] Guotao L, Xingpeng Z, Zhihui D, Huirui W (2020). SARS-CoV-2 infection presenting with hematochezia. Med Mal Infect.

[15] Wong SH, Lui RN, Sung JJ (2020). Covid-19 and the digestive system. J Gastroenterol Hepatol.

[16] Zhao D, Yao F, Wang L (2020). A comparative study on the clinical features of coronavirus 2019 (COVID-19) pneumonia with other pneumonias. Clin Infect Dis.

[17] Henry BM, de Oliveira MH, Benoit J, Lippi G (2020). Gastrointestinal symptoms associated with severity of coronavirus disease 2019 (COVID-19): a pooled analysis. Intern Emerg Med.

[18] Koperna T, Schulz F (1996). Prognosis and treatment of peritonitis. Do we need new scoring systems?. Arch Surg.

[19] Gonzálvez Guardiola P, Díez Ares JÁ, Peris Tomás N, Sebastián Tomás JC, Navarro Martínez S (2021). Intestinal perforation in patient with COVID-19 infection treated with tocilizumab and corticosteroids. Report of a clinical case. Cir Esp.

[20] Galvez A, King K, El Chaar M, Claros L (2020). Perforated marginal ulcer in a COVID-19 patient. Laparoscopy in these trying times?. Obes Surg.

[21] Scopinaro N, Gianetta E, Adami GF (1996). Biliopancreatic diversion for obesity at 18 years. Surgery.

[22] Cossu ML, Meloni GB, Alagna S, Tilocca PL, Pilo L, Profili S, Noya G (2007). Emergency surgical conditions after biliopancreatic diversion. Obes Surg.

[23] Anderson PL, Velanovich V V, Kaufman CR, Carter PL (1992). Late perforation of the distal Roux-en-Y anastomosis in a patient with biliopancreatic diversion. Obes Surg.

[24] Aabakken L, Osnes M (1989). Non-steroidal anti-inflammatory drug-induced disease in the distal ileum and large bowel. Scand J Gastroenterol Suppl.

[25] Kangas-Dick A, Prien C, Rojas K (2020). Gastrointestinal perforation in a critically ill patient with COVID-19 pneumonia. SAGE Open Med Case Rep.

[26] Hecker A, Schneck E, Röhrig R (2015). The impact of early surgical intervention in free intestinal perforation: a time-to-intervention pilot study. World J Emerg Surg.

[27] Castellví J, Pi F, Sueiras A (2011). Colonoscopic perforation: useful parameters for early diagnosis and conservative treatment. Int J Colorectal Dis.

[28] Donovan AJ, Berne TV, Donovan JA (1998). Perforated duodenal ulcer: an alternative therapeutic plan. Arch Surg.

